# Supplementary material for: Nanocomposites of Chitosan/Graphene Oxide/Titanium Dioxide Nanoparticles/Blackberry Waste Extract as Potential Bone Substitutes
Source: Polymers (Basel). 2021 Nov 10;13(22):3877. doi: 10.3390/polym13223877 (PMC8618967; doi:10.3390/polym13223877)
Supplement: Supplementary file 1 [file polymers-13-03877-s001.zip › polymers-1449099-supplementary.pdf]

Supporting information of

Nanocomposites of Chitosan/Graphene Oxide/Titanium Dioxide Nanoparticles/BlackBerry Waste Extract as Potential Bone Substitutes

Carlos Humberto Valencia Llano [1], Moisés A. Solano [2] and Carlos David Grande-Tovar [2,*]

[1] Grupo Biomateriales Dentales, Escuela de Odontología, Universidad del Valle, Calle 4B # 36-00, 76001 Cali, Colombia; carlos.humberto.valencia@correounivalle.edu.co
[2] Grupo de Investigación de Fotoquímica y Fotobiología, Facultad de Ciencias, Programa de Química, Universidad del Atlántico, Carrera 30 Número 8-49, Puerto Colombia 081008, Colombia; madolfosolano@mail.uniatlantico.edu.co
* Correspondence: carlosgrande@mail.uniatlantico.edu.co; Tel.: +57-5-3599-484

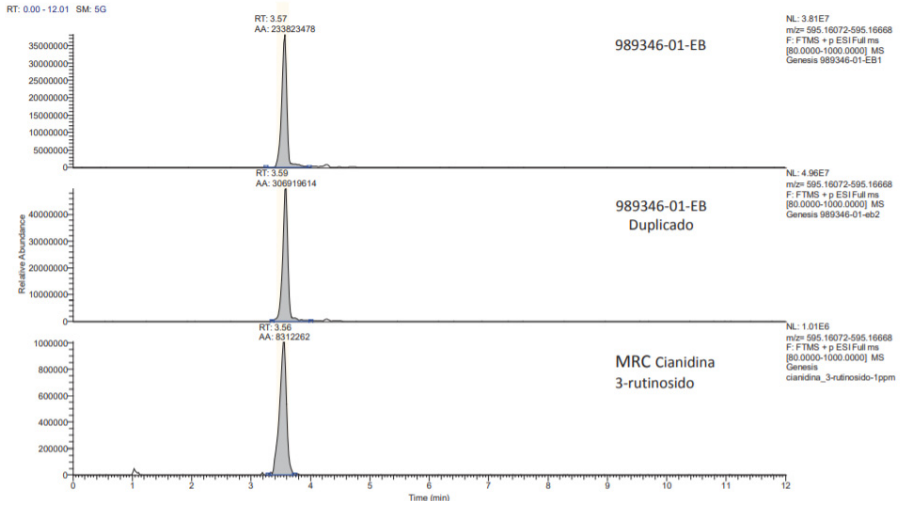

Figure S1. UHPLC chromatogram of anthocyanin cyanidin-3-rutinoside
